# Safety assessment of neurokinin-1 receptor antagonist: real-world adverse event analysis from the FAERS database

**DOI:** 10.3389/fphar.2024.1413709

**Published:** 2024-07-31

**Authors:** Chuanli Yang, Pengyang Xu, Teng Wu, Yunhe Fan, Qingqing Li, Jijun Zhang, Xiaobing Shen, Xiushan Dong

**Affiliations:** ^1^ Department of General Surgery, Shanxi Bethune Hospital, Shanxi Academy of Medical Sciences, Tongji Shanxi Hospital, Third Hospital of Shanxi Medical University, Taiyuan, China; ^2^ Key Laboratory of Environmental Medical Engineering and Education Ministry, School of Public Health, Southeast University, Nanjing, Jiangsu, China; ^3^ Department of Preventive Medicine, School of Public Health, Southeast University, Nanjing, China; ^4^ Department of Pharmacy, Shanxi Bethune Hospital, Shanxi Academy of Medical Sciences, Tongji Shanxi Hospital, Third Hospital of Shanxi Medical University, Taiyuan, China

**Keywords:** neurokinin-1 receptor antagonists, adverse events, FAERS, real-world analysis, preferred term

## Abstract

**Background:**

Aprepitant, fosaprepitant, and netupitant are three common neurokinin-1 receptor antagonists (NK-1RAs) used to prevent chemotherapy-induced nausea and vomiting, following highly or moderately emetogenic chemotherapy. Understanding their different adverse event (AE) profiles may help clinicians make appropriate treatment decisions.

**Methods:**

All data collected from the FDA Adverse Event Reporting System (FAERS) database from the first quarter of 2004 to the fourth quarter of 2023 underwent disproportionality analysis to detect, evaluate, and compare AE signals of the three NK-1RAs.

**Results:**

A total of 3,904, 1,123, and 243 AE reports related to aprepitant, fosaprepitant, and netupitant, respectively, were extracted from the FAERS database. Of these, more than 50% of respondents were female, and most of them were aged 45–65 years. General disorders and administration-site conditions, and gastrointestinal disorders were the most frequent signals in the system organ class of the three NK-1RA drugs. In addition, aprepitant was strongly associated with joint deposit (ROR = 26.27) and fosaprepitant was closely related to seizure-like phenomena (ROR = 26.90); two preferred terms (PTs) were not mentioned in the manual. Statistically, netupitant was likely to induce death (N = 63, ROR = 8.78, 95% CI: 6.75–11.42). Additionally, neutropenic colitis, colitis, and stomatitis were unique to netupitant. Furthermore, the AE profiles of the three NK-1RA drugs were different by gender.

**Conclusion:**

The AE profiles for aprepitant, fosaprepitant, and netupitant were different. In addition to paying attention to common AEs, clinicians need to pay attention to new emerging AEs, such as joint deposit, seizure-like phenomena, neutropenic colitis, colitis, and stomatitis, regarding the three NK-1RA drugs. Furthermore, the AE compositions of the three NK-1RA drugs were different in different genders, and clinicians should take these factors into account when selecting NK-1RAs for CINV treatment.

## 1 Introduction

Chemotherapy-induced nausea and vomiting (CINV) is a common side effect that significantly impacts the quality of life and treatment adherence of cancer patients (Barbour, 2012). Its occurrence involves complex regulation of multiple neurotransmitters and receptors (Gupta et al., 2021). Neurokinin-1 receptor antagonists (NK-1RAs) represent a class of medications that exert antiemetic effects by blocking central and peripheral NK-1 receptors, thereby inhibiting the release of substance P (SP) (Karthaus et al., 2019; Pojawa-Gołąb et al., 2019). These agents not only demonstrate anti-emetic properties but also exhibit unique anxiolytic and antidepressant effects. Combination therapy with 5-hydroxytryptamine 3 (5-HT3) receptor antagonists and/or corticosteroids with NK-1RAs is a common strategy recommended by guidelines such as those from the National Comprehensive Cancer Network (NCCN) and the American Society of Clinical Oncology (ASCO) for managing CINV induced by moderately and highly emetogenic chemotherapy regimens (Hesketh et al., 2020).

Among the three main NK-1RAs used clinically, aprepitant, fosaprepitant, and netupitant, each demonstrates significant efficacy in the prevention and alleviation of CINV. Aprepitant, the first NK-1RA approved by the FDA in 2003, is widely utilized for the prophylaxis of CINV in cancer patients (Aapro et al., 2015). Typically, 125 mg of aprepitant was given orally on day 1 (1 h before chemotherapy), followed by 80 mg on days 2 and 3 for adults. Additionally, aprepitant was used in combination with dexamethasone and ondansetron (Poli-Bigelli et al., 2003). Fosaprepitant, a phosphorylated prodrug of aprepitant, is administered intravenously and rapidly metabolizes into aprepitant to exert antiemetic effects. It received the FDA approval in 2008 (Hale et al., 2000; Xue et al., 2023). A single intravenous dose of 150 mg of fosaprepitant is an effective and globally well-tolerated supplement to an antiemetic regimen that includes dexamethasone and a 5-HT3 receptor antagonist (Garnock-Jones, 2016). Netupitant, a novel generation NK-1RA, possesses higher selectivity and provides prolonged antiemetic effects. It is commonly co-administered with palonosetron and used in the treatment of CINV (Rojas and Slusher, 2015). Hesketh PJ et al. indicated that the combination drug of 300 mg of netupitant with 0.5 mg of palonosetron was the optimal combination dose with good safety and tolerability (Hesketh et al., 2014).

Although all three of these NK-1RAs have demonstrated promising efficacy in controlling nausea and vomiting, clinical decisions should not be solely based on efficacy. The safety and tolerability profiles of these agents are equally important. Concerns have been raised regarding aprepitant-induced adverse events, notably cases of aprepitant-associated neurological toxicity, which have been observed in a real-world study (Kataria et al., 2017). Additionally, common adverse reactions associated with aprepitant treatment include headache, fatigue, anorexia, constipation, diarrhea, nausea, and hiccups (Larusso et al., 2008). For fosaprepitant, common adverse events observed in clinical trials include headache, dizziness, asthenia, abdominal pain, diarrhea, constipation, anorexia, and hiccups (Colon-Gonzalez and Kraft, 2010; Maru et al., 2013). Moreover, due to differences in administration routes, fosaprepitant has been associated with immediate hypersensitivity reactions, including flushing, erythema, and dyspnea, during infusion. In real-life scenarios, concerns have been raised about netupitant-induced serotonin syndrome, which can be life-threatening. In addition, the most commonly reported adverse events with netupitant include headache, constipation, and fatigue (Shirley, 2021).

The FDA Adverse Event Reporting System (FAERS) is a public database, which is aimed at supporting the post-market safety monitoring of drugs and therapeutic biologic products through spontaneous reports from consumers, healthcare professionals, drug manufacturers, and other non-healthcare providers (Ma et al., 2022). In this study, we utilized the FAERS database to compare adverse event reports associated with aprepitant, fosaprepitant, and netupitant. This research aims to provide real-world evidence to better understand the balance between efficacy and toxicity of NK-1RAs. Importantly, it may inform the management of aprepitant, fosaprepitant, and netupitant therapies based on real-world evidence.

### 1.1 Data sources

Considering the time to market of the three NK-1RA drugs, American Standard Code for Information Interchange (ASCII) report files were downloaded from the FAERS database from the 1st quarter of 2004 to the 4th quarter of 2023 for this study. The data were loaded into MySQL 15.0 and handled using Navicat Premium 15 software.

### 1.2 Data extraction and analysis

Duplicate reports were dropped. For DEMO table data with the same case id, only the latest report was retained based on the date. The primary id field was used to establish relationships between datasets and to correct for age and weight indicator anomalies. The standardization of drug names was performed through the Medex_UIMA_1.8.3 system. Reports, where the primary drug suspected to be associated with AEs was three NK-1RA drugs, were extracted. These reports included a variety of information, such as the date of the report, age, and sex of the patient, reporter, and region.

In our study, four disproportionality methods, namely, the reported odds ratio (ROR), the proportional reporting ratio (PRR), the Bayesian confidence propagation neural network (BCPNN), and the empirical Bayesian geometric mean (EBGM), were used to detect drug AE signals. Each of the four algorithms possesses distinct advantages: ROR has the strength of correcting bias due to the low number of reports for certain events, PRR has the advantage of higher specificity than ROR, BCPNN is good at combining and cross-validating data from multiple sources, and EBGM excels in detecting signals from rare events. In this study, a combination of ROR, PRR, BCPNN, and EBGM algorithms was used to expand the detection range, validate the results from multiple aspects by taking advantages of each algorithm, and rationally utilize the distinctive characteristics of different approaches to identify more reliable safety signals. The combined use of multiple algorithms provides cross-validation to minimize false positives, and by adjusting thresholds and variances, more potentially rare adverse reactions can be detected. All algorithms are based on a 2 × 2 contingency table (Supplementary Table S1). Specific formulas and cutoff thresholds are shown in Supplementary Table S1, and statistical analyses were performed using R software. Higher values indicated stronger signal strength, suggesting a more robust association between the target drug and the adverse event.

### 1.3 Signal filtering and categorization

Preferred terms (PTs) with reported counts ≥3 were selected for in the initial screening of this study. The Medical Dictionary for Regulatory Activities (MedDRA) was employed to encode, categorize, and localize signals, thus facilitating the analysis of the specific system organ class (SOC) involved in the signal of an adverse event.

## 2 Results

### 2.1 Basic characteristics of neurokinin-1 receptor antagonist-related adverse drug events

**FIGURE 1:**
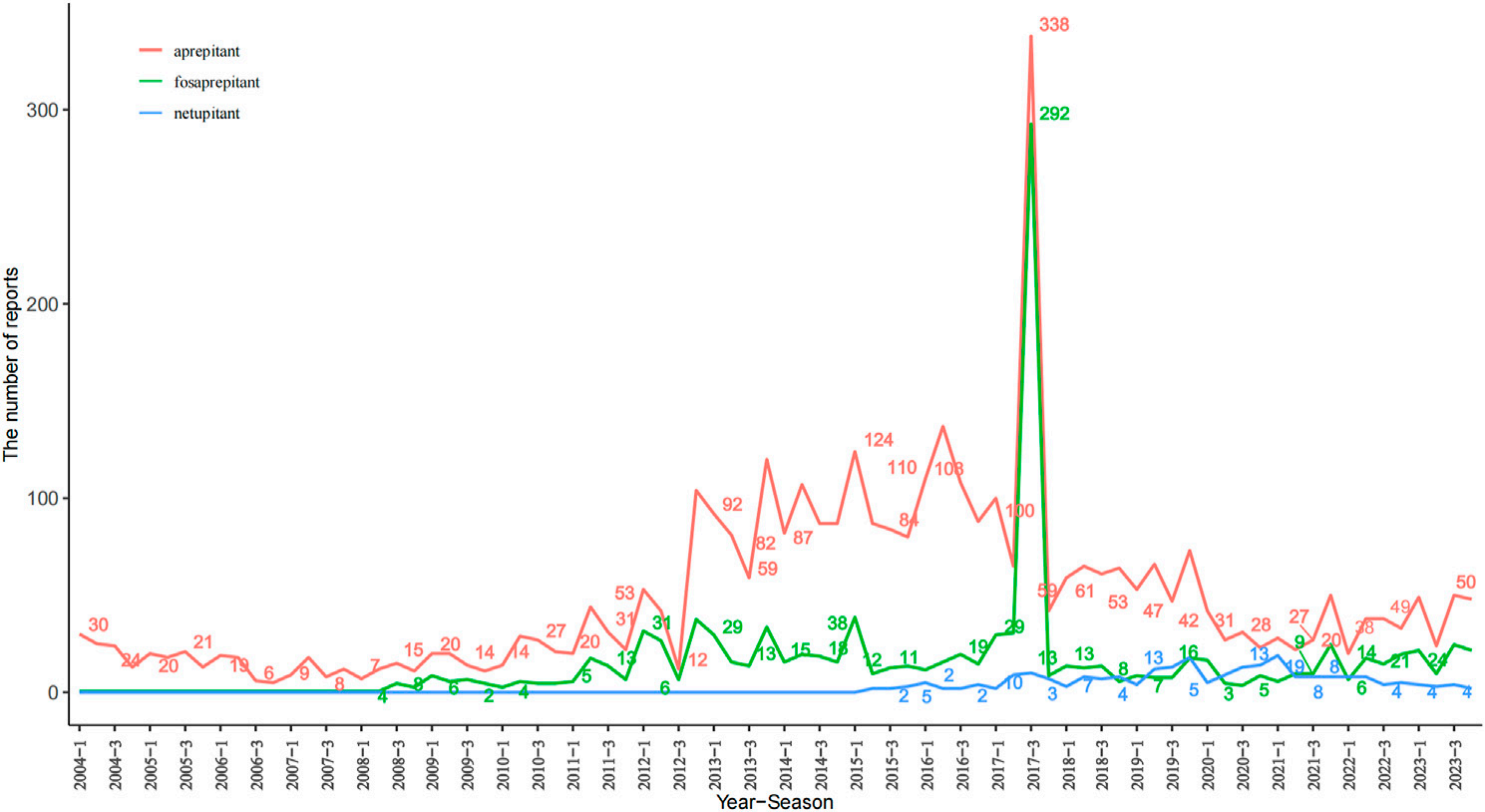
Quarterly number of adverse events reported post marketing for aprepitant, fosaprepitant, and netupitant. The x-axis represents the timeline of drug use, and the y-axis shows the number of reports per quarter.

Between Q1 of 2004 and Q4 of 2023, a total of 16,800,135 AE reports were collected from the FAERS, of which 3,904 were associated with aprepitant, 1,123 with fosaprepitant, and 243 with netupitant. Aprepitant was approved for use in 2003, with AE reports peaking at 545 in 2017 (Q3, AE reports = 338). The use of fosaprepitant began in 2008, and it already reached 359 AE reports in 2017 (Q3, AE reports = 292). Netupitant was put into marketed use in 2015 with an AER of less than 50 per year, as shown in Figure 1.

The majority of the reports came from individuals aged 45–65 years, but a considerable proportion of unknown cases were identified. Female respondents reported 53.46%, 51.47%, and 58.02% of AE reports for aprepitant, fosaprepitant, and netupitant, respectively, (Table 1). Meanwhile, in males, 32.68%, 36.78%, and 38.68 AE reports were for aprepitant, fosaprepitant, and netupitant, respectively. Most of these reports originated in the United States, and then in France and other countries such as South Korea and Japan. Notably, reports from physicians ranked first rather than consumers, in all three medications, adding credibility to our study. Except for unknown serious medical events, hospitalization was the most commonly reported serious adverse outcome in three medications, followed by death in aprepitant (10.61%) and netupitant (31.03%), and life-threatening conditions in fosaprepitant (8.57%). Disability, required intervention to prevent permanent impairment/damage, and congenital anomalies were included; the numbers were marginal.

**TABLE 1:** Basic characteristics of AE reports related to three NK-1RAs in the FAERS database.

Variable	Aprepitant, N (%)	Fosaprepitant, N (%)	Netupitant, N (%)
Age (years)			
<18	103 (2.64)	21 (1.87)	1 (0.41)
18–45	452 (11.58)	152 (13.54)	28 (11.52)
45–65	1,197 (30.66)	426 (37.93)	102 (41.98)
65–75	608 (15.57)	201 (17.90)	54 (22.22)
≥75	203 (5.20)	70 (6.23)	14 (5.76)
Unknown	1,341 (34.35)	253 (22.53)	44 (18.11)
Sex			
Female	2,087 (53.46)	578 (51.47)	141 (58.02)
Male	1,276 (32.68)	413 (36.78)	94 (38.68)
Unknown	541 (13.86)	132 (11.75)	8 (3.29)
Reporter			
Physician	1,146 (29.35)	451 (40.16)	95 (39.09)
Consumer	1,072 (27.46)	203 (18.08)	29 (11.93)
Other health professional	816 (20.90)	170 (15.14)	43 (17.70)
Pharmacist	794 (20.34)	280 (24.93)	55 (22.63)
Lawyer	1 (0.03)		1 (0.41)
Registered nurse	1 (0.03)		
Unknown	74 (1.90)	19 (1.69)	20 (8.23)
Reported countries			
United States	1,778 (45.54)	409 (36.42)	122 (50.21)
France	399 (10.22)		
Korea, South	347 (8.89)	338 (30.10)	
Japan	246 (6.30)	113 (10.06)	
Other	1,134 (29.05)	263 (23.42)	121 (49.79)
Outcomes			
Hospitalization	1,095 (31.75)	290 (24.85)	76 (32.76)
Death	366 (10.61)	73 (6.26)	72 (31.03)
Life-threatening	299 (8.67)	100 (8.57)	5 (2.16)
Disability	120 (3.48)	32 (2.74)	6 (2.59)
Required intervention to prevent permanent impairment/damage	22 (0.64)	17 (1.46)	
Congenital anomaly	1 (0.03)		1 (0.43)
Unknown	1,546 (44.82)	655 (56.13)	72 (31.03)

### 2.2 Disproportionality analyses associated with aprepitant, fosaprepitant, and netupitant

#### 2.2.1 Detection of signals at the system organ class level

As shown in Table 2, the occurrence of aprepitant-, fosaprepitant-, and netupitant-induced ADRs was mainly directed to 24, 21, and 15 SOCs, respectively. Some of these findings matched the SOCs corresponding to common adverse reactions in the drug inserts, indicating a solid reliability of the data. Among them, the SOCs with more AEs were general disorders and administration-site conditions (aprepitant, N = 2,346; fosaprepitant, N = 837; and netupitant, N = 143), and they were powerfully positive in all four algorithms (Supplementary Table S2). Notably, some of the apparent AE-involved SOCs were similar between aprepitant and fosaprepitant, but netupitant was apparently inconsistent with the SOCs of the two previously mentioned NK-1RA drugs. Especially in benign, malignant, and unspecified neoplasms (including cysts and polyps), the number of AE reports was 190 (1.65%) and 29 (0.75%) for aprepitant and fosaprepitant, respectively; however, netupitant was not detected.

**TABLE 2:** SOCs associated with case reports of aprepitant, fosaprepitant, and netupitant adverse events.

SOC	Aprepitant, N (%)	Fosaprepitant, N (%)	Netupitant, N (%)
General disorders and administration-site conditions	2,346 (20.41)	837 (21.56)	143 (27.93)
Gastrointestinal disorders	1,400 (12.18)	477 (12.28)	111 (21.68)
Respiratory, thoracic, and mediastinal disorders	942 (8.19)	408 (10.51)	21 (4.10)
Nervous system disorders	900 (7.83)	232 (5.97)	51 (9.96)
Investigations	747 (6.50)	232 (5.97)	24 (4.69)
Skin and subcutaneous tissue disorders	685 (5.96)	211 (5.43)	17 (3.32)
Injury, poisoning, and procedural complications	656 (5.71)	180 (4.64)	26 (5.08)
Vascular disorders	593 (5.16)	262 (6.75)	10 (1.95)
Musculoskeletal and connective tissue disorders	538 (4.68)	171 (4.40)	6 (1.17)
Immune system disorders	395 (3.44)	145 (3.73)	5 (0.98)
Blood and lymphatic system disorders	395 (3.44)	159 (4.09)	24 (4.69)
Infections and infestations	391 (3.40)	177 (4.56)	17 (3.32)
Metabolism and nutrition disorders	314 (2.73)	114 (2.94)	13 (2.54)
Psychiatric disorders	298 (2.59)	77 (1.98)	18 (3.52)
Cardiac disorders	245 (2.13)	77 (1.98)	26 (5.08)
Benign, malignant, and unspecified neoplasms (including cysts and polyps)	190 (1.65)	29 (0.75)	-
Hepatobiliary disorders	133 (1.16)	16 (0.41)	-
Renal and urinary disorders	127 (1.10)	36 (0.93)	-
Eye disorders	95 (0.83)	24 (0.62)	-
Endocrine disorders	33 (0.29)	9 (0.23)	-
Ear and labyrinth disorders	28 (0.24)	-	-
Reproductive system and breast disorders	20 (0.17)	10 (0.26)	-
Congenital, familial, and genetic disorders	16 (0.14)	-	-
Pregnancy, puerperium, and perinatal conditions	8 (0.07)	-	-

#### 2.2.2 Detection of signals at preferred term levels

To improve specificity and reduce the probability of miscategorization, we ranked the preferred terms (PT) in descending order based on the ROR value. The top 19 for aprepitant, top 20 for fosaprepitant, and top 19 for netupitant, classified as SOCs, are shown in Table 3. The PRR, BCPNN, and EBGM results are presented in Supplementary Table S3. In the SOC of general disorders and administration-site conditions, the main PTs were site-of-administration-related, in aprepitant and fosaprepitant. Statistically, the tendency of aprepitant (ROR = 45.32, 95% CI: 34.08–60.26) and fosaprepitant (ROR = 44.91, 95% CI: 27.02–74.63) to induce phlebitis was comparable in vascular disorders of SOCs. Additionally, the signal of vein discoloration was only present in aprepitant (ROR = 51.37, 95% CI: 21.26–124.1). In the respiratory, thoracic, and mediastinal disorders, aprepitant and fosaprepitant had strong hiccup signals. In the injury, poisoning, and procedural complication systems, both drugs showed strong signals of radiation esophagitis, but the ROR comparison showed that fosaprepitant (ROR = 209.74, 95% CI: 86.57–508.17) had a higher ROR than aprepitant (ROR = 39.61, 95% CI: 30.83–50.9). Similarly, infusion-related hypersensitivity reaction was more likely to occur in fosaprepitant (ROR = 114.27, 95% CI: 36.63–356.43) than in aprepitant (ROR = 41.59, 95% CI: 13.34–129.7) in immune system disorders, as shown in Table 3.

**TABLE 3:** Adverse event signaling comparison of aprepitant, fosaprepitant, and netupitant at the preferred term level.

SOC	PT	Case report	Aprepitant ROR (95% CI)	Case report	Fosaprepitant ROR (95% CI)	Case report	Netupitant ROR (95% CI)
General disorders and administration-site conditions	Injection-site vasculitis	13	1415.4 (755.37, 2652.19)	13	3997.74 (2127.99, 7510.33)		
	Injection-site phlebitis	28	610.51 (410.66, 907.62)	27	2083.27 (1381.81, 3140.84)		
	Infusion-site phlebitis	8	170.63 (84.14, 346.01)	3	190.04 (60.7, 594.93)		
	Infusion-site irritation	18	89.75 (56.25, 143.19)	9	123.6 (64.04, 238.55)		
	Infusion-site urticaria	8	72.71 (36.14, 146.29)	5	127.03 (52.59, 306.83)		
	Infusion-site rash	11	46.42 (25.62, 84.12)	7	83.94 (39.88, 176.66)		
	Infusion-site reaction	19	44.62 (28.38, 70.14)	14	103.44 (61.06, 175.22)		
	Infusion-site pain	81	36.12 (29.01, 44.99)	41	51.11 (37.54, 69.57)		
	Infusion-site discomfort	5	30.35 (12.59, 73.17)	4	66.62 (24.92, 178.09)		
	Infusion-site induration			3	38.77 (12.47, 120.48)		
	Injection-site atrophy			4	35.46 (13.28, 94.68)		
	Injection-site induration			31	33.55 (23.55, 47.79)		
	Infusion-site extravasation			13	28.44 (16.49, 49.06)		
	Infusion-site pruritus			5	27.62 (11.48, 66.46)		
	Disease progression					19	18.99 (12.01, 30.03)
	Drug interaction					15	11.58 (6.93, 19.35)
	Death					63^a^	8.78 (6.75, 11.42)
Vascular disorders	Vein discoloration	5	51.37 (21.26, 124.1)				
	Phlebitis	48	45.32 (34.08, 60.26)	15	44.91 (27.02, 74.63)		
Benign, malignant, and unspecified neoplasms (including cysts and polyps)	Testicular germ cell cancer metastatic	3	553.37 (166.13, 1843.3)				
	Seminoma	3^a^	62.09 (19.86, 194.13)				
Investigations	Computerized tomogram thorax abnormal	4	26.27 (9.83, 70.22)				
	Pulse pressure decreased	3	25.76 (8.28, 80.17)				
Respiratory, thoracic, and mediastinal disorders	Hiccups	62	39.61 (30.83, 50.9)	20	39.67 (25.55, 61.61)		
Musculoskeletal and connective tissue disorders	Joint deposit	3^a^	303.04 (93.91, 977.82)				
Injury, poisoning, and procedural complications	Radiation esophagitis	5	63.9 (26.42, 154.58)	5	209.74 (86.57, 508.17)		
Immune system disorders	Infusion-related hypersensitivity reaction	3	41.59 (13.34, 129.7)	3	114.27 (36.63, 356.43)		
Nervous system disorders	Seizure-like phenomena			3^a^	26.9 (8.66, 83.54)		
Infections and infestations	Injection-site cellulitis			5	42.16 (17.51, 101.51)		
Gastrointestinal disorders	Neutropenic colitis					7^a^	375.78 (177.88, 793.86)
	Colitis					4^a^	12.04 (4.5, 32.21)
	Stomatitis					5^a^	8.92 (3.69, 21.51)
	Constipation					11	5.83 (3.21, 10.6)
	Vomiting					21	5.58 (3.6, 8.63)
	Nausea					29	4.4 (3.03, 6.4)
Blood and lymphatic system disorders	Leukopenia					5	12.11 (5.02, 29.21)
	Neutropenia					9	7.1 (3.67, 13.72)
	Febrile neutropenia					4	6.9 (2.58, 18.45)
Nervous system disorders	Serotonin syndrome					4	26.33 (9.84, 70.44)
	Encephalopathy					5	24.75 (10.26, 59.72)
Metabolism and nutrition disorders	Hyponatremia					5^a^	10.71 (4.44, 25.85)
	Dehydration					5^a^	4.76 (1.97, 11.47)
Vascular disorders	Flushing					4^a^	5.86 (2.19, 15.66)
Infections and infestations	Septic shock					3^a^	8.27 (2.66, 25.73)
Cardiac disorders	Bradycardia					4^a^	8.9 (3.33, 23.79)

^a^ indicates the PT that does not appear in the instructions.

Some signals were unique to aprepitant and were distributed in the following SOC: in benign, malignant, and unspecified (including cysts and polyps) neoplasms, testicular germ cell carcinoma metastases (ROR = 51.37, 95% CI: 21.26–124.1) and seminoma (ROR = 45.32, 95% CI: 34.08–60.26) were unique safety signals. In the investigations, the signal of a computerized tomogram thorax abnormal was strong (ROR = 26.27, 95% CI: 9.83–70.22), which was not mentioned in the instructions. Joint deposits in musculoskeletal and connective tissue disorders were, likewise, unique safety signals of aprepitant. For fosaprepitant, in addition to the common adverse reactions at the site of administration in the two SOCs of general disorders and administration-site conditions, as well as infections and infestations, attention should be paid to its seizure-like phenomena and adverse (ROR = 26.9, 95% CI: 8.66–83.54) events in the nervous system.

Although three drugs are neurokinin-1 receptor antagonists, netupitant exhibits dramatic differences in AE signals compared to aprepitant or fosaprepitant. In the gastrointestinal system, neutropenic colitis (ROR = 375.78, 95% CI: 177.88–793.86) and stomatitis (ROR = 8.92, 95% CI: 3.69–21.51) were unique safety signals that were not mentioned in the instructions. Statistically, netupitant was likely to induce death (N = 63, ROR = 8.78, 95% CI: 6.75–11.42) in general disorders and administration-site conditions of SOC. Furthermore, hyponatremia (ROR = 10.71, 95% CI: 4.44–25.85) and dehydration (ROR = 4.76, 95% CI: 1.97–11.47) in metabolism and nutritional disorders were common PT to netupitant, similar to flushing (ROR = 5.86, 95% CI: 2.19–15.66), septic shock (ROR = 8.27, 95% CI: 2.66–25.73), and bradycardia (ROR = 8.9, 95% CI: 3.33, 23.79), which were identified as strong signals of netupitant in vascular disorders, infections and infestations, and cardiac disorders of SOC, respectively.

#### 2.2.3 Comparison of safety signals in four system organ classes

**FIGURE 2:**
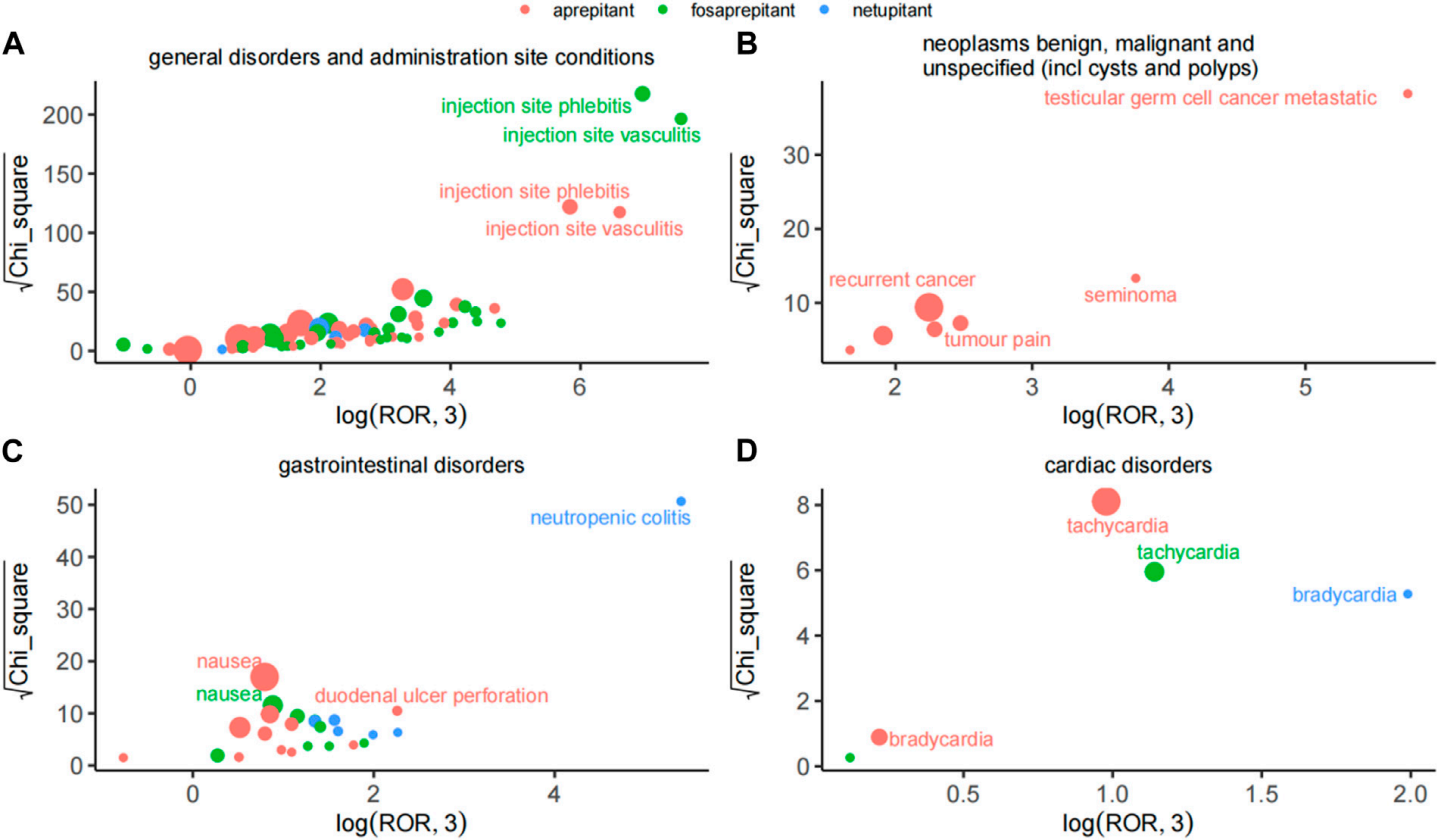
Comparison of safety signals of the three neurokinin-1 receptor antagonists in four system organ classes. **(A)** General disorders and administration-site conditions. **(B)** Benign, malignant, and unspecified neoplasms (including cysts and polyps). **(C)** Gastrointestinal disorders. **(D)** Cardiac disorders. The size of the dot indicates the number of adverse reactions reported.

Considering the severity and specificity of PT, we compared the AE signals in the four SOCs and observed that the AE signals of the three drugs had their features, as shown in Figure 2. In general disorders and administration-site conditions, injection-site phlebitis and injection-site vasculitis were the strongest signals associated with aprepitant and fosaprepitant, as indicated by ROR and Chi_square. Among benign, malignant, and unspecified neoplasms (including cysts and polyps), testicular germ cell cancer metastatic was the most potent AE in aprepitant, and recurrent cancer was the most reported event also in aprepitant. Notably, the netupitant’s strongest signal was neutropenic colitis in the gastrointestinal disorder SOC based on the ROR and Chi_square mined. Finally, in cardiac disorders of SOC, we observed that the dominant PT for aprepitant and fosaprepitant was tachycardia, whereas the strongest signal for aprepitant was bradycardia, with minimal differences in both ROR and Chi_square.

#### 2.2.4 Sex scans of safety signals

**FIGURE 3:**
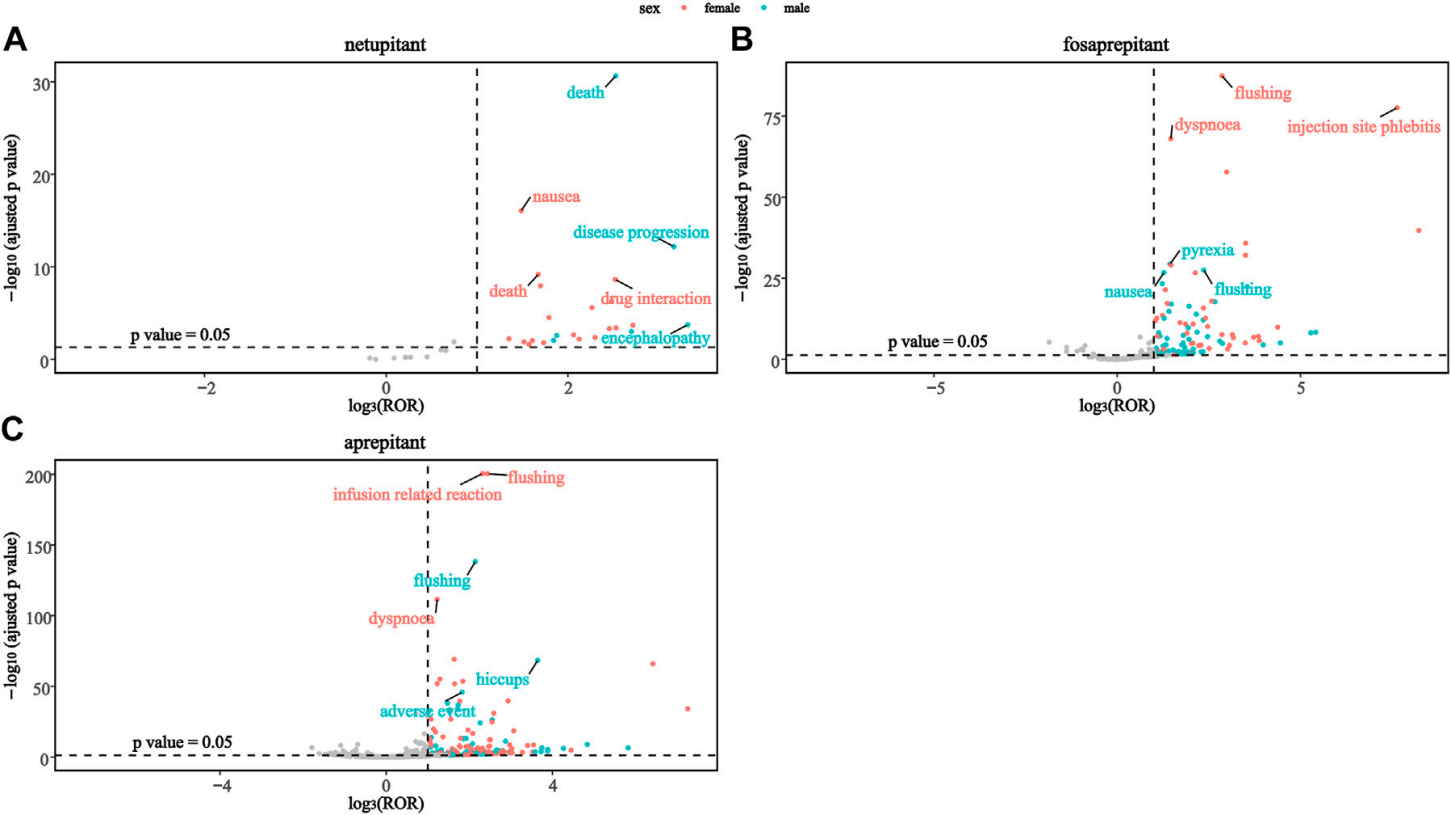
Comparison of both sex safety signals among three neurokinin-1 receptor antagonists: **(A)** netupitant, **(B)** fosaprepitant, and **(C)** aprepitant.

To investigate the changes in each signal in different genders, this study mapped the sex scans of the safety signals of the three drugs. As shown in Figure 3A, death was a stronger positive signal in males than in females in the netupitant. In addition, the second and third significant signals were different in netupitant, encephalopathy, and disease progression in males, and drug interaction and nausea in females. In fosaprepitant and aprepitant, both sexes showed strong signals of flushing, but females showed a higher signal than males. Moreover, in female users of aprepitant and fosaprepitant, the positive signals for infusion-related reaction and injection-site phlebitis were stronger than in males compared to females (Figure 3). In addition, dyspnea signals were more common in women taking fosaprepitant and aprepitant.

## 3 Discussion

Disproportionality analysis based on the FAERS database revealed that AE profiles were different among three NK-1RAs. Specifically, aprepitant and fosaprepitant demonstrated consistent signals across multiple SOCs, primarily concentrated in the general disorders and administration-site conditions. On the other hand, netupitant exhibited unique signals in the gastrointestinal disorders and metabolism and nutritional disorders of SOCs. These differences may be attributed to the variations in the chemical structure, metabolic pathways, pharmacokinetic properties, receptor affinity characteristics, and combination therapy among the three drugs. Understanding these molecular mechanisms can help optimize drug selection and manage adverse reactions effectively.

A higher occurrence and association of AEs such as injection-site vasculitis and injection-site phlebitis were noted with fosaprepitant. This could be attributed to the presence of non-ionic surfactant polysorbate 80 in fosaprepitant for injection (Boccia et al., 2019). Polysorbate 80 is a biologically active compound which is widely used in various intravenous formulations, such as docetaxel and recombinant human erythropoietin injection (Garnock-Jones, 2016). Injection and infusion-site adverse events (ISAEs), such as allergic reactions, injection-site pain, erythema, and thrombophlebitis, may partially result from the presence of polysorbate 80 (Boccia et al., 2019). Clinical trials have shown a higher incidence of ISAEs with fosaprepitant than aprepitant (Schwartzberg and Navari, 2018). A retrospective study suggests that fosaprepitant infusion via a central venous catheter reduces the incidence of ISAEs in breast cancer patients (Tsuda et al., 2016). It is suggested that changing the way the infusion is administered may reduce fosaprepitant infusion-site-related adverse effects.

Additionally, some studies have suggested the occurrence of neurotoxicity events reported with the combination of aprepitant/fosaprepitant and ifosfamide (Durand et al., 2007; Kataria et al., 2017; Vazirian et al., 2022). However, there were also studies that have noted no statistically significant association between ifosfamide-induced neurotoxicity and aprepitant administration (Szabatura et al., 2015; Modi and Cimino, 2021; Vazirian et al., 2022). To explain the occurrence of associated neurotoxic AEs, it has been suggested that the pharmacokinetics of ifosfamide concentrations are significantly altered due to the inhibition of the CYP3A4 concomitant administration of aprepitant or fosaprepitant (Durand et al., 2007; Séjourné et al., 2014). However, a study evaluating the effect of aprepitant on the pharmacokinetics of ifosfamide showed that no substantial changes in ifosfamide concentrations were detected after the administration of aprepitant (Xiong et al., 2019). In our study, fosaprepitant was found to be associated with seizure-like phenomena adverse events (ROR = 26.9, 95% CI: 8.66–83.54), which suggests that patients’ neurological symptoms should be closely monitored while using fosaprepitant, and if necessary, an epileptiform electroencephalography is suggested. In conclusion, further studies and larger data are needed to clinically validate the association between NK-1RA and ifosfamide-induced neurotoxicity.

Netupitant/palonosetron is a novel fixed-dose combination, comprising 300 mg netupitant and 0.50 mg palonosetron, approved in the US and the EU for preventing CINV in adults (Keating, 2015). A phase III clinical study evaluating its efficacy and safety in preventing CINV showed that the most common adverse events were headache and constipation, without related AE, leading to discontinuation or death (Aapro et al., 2014). However, our study found a statistically significant likelihood of death (N = 63, ROR = 8.78, 95% CI: 6.75–11.42) associated with netupitant in the general disorders and administration-site conditions of SOC. Furthermore, netupitant showed a strong signal with neutropenic colitis and colitis, which are not listed as drug instructions. This may be related to substance P (SP) activation or another component of palonosetron. Cancer patients undergoing chemotherapy or other immunosuppressive treatments often experience decreased neutrophil levels. Therefore, when using netupitant to prevent CINV, enhanced monitoring and management of neutropenia should be emphasized. To date, there have been few reported cases of adverse events with netupitant/palonosetron, and further research and clinical data are needed to draw definitive results.

NK-1RA is considered a promising therapy for tendinopathy, rheumatoid arthritis, and osteoarthritis (Ko et al., 2022). SP is a neuro-inflammatory mediator produced by sensory nerve fibers and local inflammatory cells, playing a crucial role in various physiological and pathological functions such as inflammation, angiogenesis, and pain (Janelsins et al., 2013; Ko et al., 2022). Aberrant expression of the SP–NK1R pathway in various inflammatory diseases suggests its involvement in the inflammatory process. Our study results indicated a strong association of joint deposits with aprepitant, which was not mentioned in drug’s labeling and possibly related to the activation of the SP–NK1R pathway. Joint deposit is usually associated with the inflammatory response, accumulation of metabolites, or other pathological substances around the joints; therefore, special attention should be paid to the presence of patients at risk for gout, hemochromatosis, and chronic inflammatory diseases when using aprepitant for CINV. Additionally, there were significantly more reports from female patients than male patients, indicating a potential gender-specific occurrence of AEs with NK-1RAs. For instance, male patients showed a higher frequency of death with aprepitant, whereas females exhibited higher signals for infusion-related reactions and injection-site phlebitis. These sex-specific differences underscore the importance of gender consideration in pharmacovigilance and personalized medicine approaches.

This study provides post-marketing AE signal analysis based on real-world data with a large sample size, which is crucial for evaluating drug safety risks. However, it has certain limitations. First, the FAERS database is a self-reporting system, leading to potential inaccuracies, under-reporting biases, and reporting biases, which may affect study results. Second, although methods such as ROR, PRR, BCPNN, and EBGM are commonly used for identifying drug adverse signals and reducing data biases, false-positive signals may still occur. Third, due to the limitations of the database, information on the entire population receiving the drug is lacking, making it impossible to calculate the incidence rate of adverse events related to the target drug NK-1RA. Moreover, the FAERS database has a greater proportion of data from the United States and fewer AE reports from other countries, resulting in an inherent bias in the dataset. The identified adverse event signals only demonstrate the statistical association between NK-1RA and these events, but high-quality clinical trial research is still needed for clinical decision-making.

## 4 Conclusion

Among three NK-1RAs, the AE profiles for aprepitant, fosaprepitant, and netupitant were different. Joint deposits and seizure-like phenomena were AE signals specific to aprepitant and fosaprepitant, respectively, and they were not described in the instructions. Netupitant-induced neutropenic colitis, colitis, stomatitis, and death should be given enough attention, and its long-term effects remain unknown. Furthermore, the AE compositions of the three NK-1RA drugs were different in different genders, and clinicians should take these factors into account when selecting NK-1RAs for CINV treatment, with an emphasis on a personalized medical therapy approach.

## Data Availability

The datasets presented in this study can be found in online repositories. The names of the repository/repositories and accession number(s) can be found below: https://www.fda.gov/drugs/drug-approvals-and-databases/fda-adverse-event-reporting-system-faers.

## References

[B1] Aapro M. Carides A. Rapoport B. L. Schmoll H.-J. Zhang L. Warr D. (2015). Aprepitant and fosaprepitant: A 10-year review of efficacy and safety. Oncol. 20 (4), 450–458. 10.1634/theoncologist.2014-0229 PMC4391760 25795636

[B2] Aapro M. Rugo H. Rossi G. Rizzi G. Borroni M. E. Bondarenko I. (2014). A randomized phase III study evaluating the efficacy and safety of NEPA, a fixed-dose combination of netupitant and palonosetron, for prevention of chemotherapy-induced nausea and vomiting following moderately emetogenic chemotherapy. Ann. Oncol. 25 (7), 1328–1333. 10.1093/annonc/mdu101 24603643 PMC4071754

[B3] Barbour S. Y. (2012). Corticosteroids in the treatment of chemotherapy-induced nausea and vomiting. J. Natl. Compr. Cancer Netw. JNCCN 10 (4), 493–499. 10.6004/jnccn.2012.0049 22491048

[B4] Boccia R. Geller R. B. Clendeninn N. Ottoboni T. (2019). Hypersensitivity and infusion-site adverse events with intravenous fosaprepitant after anthracycline-containing chemotherapy: a retrospective study. Future Oncol. Lond. Engl. 15 (3), 297–303. 10.2217/fon-2018-0662 30301373

[B5] Colon-Gonzalez F. Kraft W. K. (2010). Pharmacokinetic evaluation of fosaprepitant dimeglumine. Expert Opin. Drug Metabolism Toxicol. 6 (10), 1277–1286. 10.1517/17425255.2010.513970 PMC3155701 20795794

[B6] Durand J. P. Gourmel B. Mir O. Goldwasser F. (2007). Antiemetic neurokinin-1 antagonist aprepitant and ifosfamide-induced encephalopathy. Ann. Oncol. 18 (4), 808–809. 10.1093/annonc/mdm104 17389531

[B7] Garnock-Jones K. P. (2016). Fosaprepitant dimeglumine: a review in the prevention of nausea and vomiting associated with chemotherapy. Drugs 76 (14), 1365–1372. 10.1007/s40265-016-0627-7 27510503

[B8] Gupta K. Walton R. Kataria S. P. (2021). Chemotherapy-induced nausea and vomiting: pathogenesis, recommendations, and new trends. Cancer Treat. Res. Commun. 26, 100278. 10.1016/j.ctarc.2020.100278 33360668

[B9] Hale J. J. Mills S. G. MacCoss M. Dorn C. P. Finke P. E. Budhu R. J. (2000). Phosphorylated morpholine acetal human neurokinin-1 receptor antagonists as water-soluble prodrugs. J. Med. Chem. 43 (6), 1234–1241. 10.1021/jm990617v 10737756

[B10] Hesketh P. J. Kris M. G. Basch E. Bohlke K. Barbour S. Y. Clark-Snow R. A. (2020). Antiemetics: ASCO guideline update. J. Clin. Oncol. 38 (24), 2782–2797. 10.1200/JCO.20.01296 32658626

[B11] Hesketh P. J. Rossi G. Rizzi G. Palmas M. Alyasova A. Bondarenko I. (2014). Efficacy and safety of NEPA, an oral combination of netupitant and palonosetron, for prevention of chemotherapy-induced nausea and vomiting following highly emetogenic chemotherapy: a randomized dose-ranging pivotal study. Ann. Oncol. 25 (7), 1340–1346. 10.1093/annonc/mdu110 24608196 PMC4071755

[B12] Janelsins B. M. Sumpter T. L. Tkacheva O. A. Rojas-Canales D. M. Erdos G. Mathers A. R. (2013). Neurokinin-1 receptor agonists bias therapeutic dendritic cells to induce type 1 immunity by licensing host dendritic cells to produce IL-12. Blood 121 (15), 2923–2933. 10.1182/blood-2012-07-446054 23365459 PMC3624938

[B13] Karthaus M. Schiel X. Ruhlmann C. H. Celio L. (2019). Neurokinin-1 receptor antagonists: review of their role for the prevention of chemotherapy-induced nausea and vomiting in adults. Expert Rev. Clin. Pharmacol. 12 (7), 661–680. 10.1080/17512433.2019.1621162 31194593

[B14] Kataria P. S. Kendre P. P. Patel A. A. (2017). Ifosfamide-induced encephalopathy precipitated by aprepitant: a rarely manifested side effect of drug interaction. J. Pharmacol. Pharmacother. 8 (1), 38–40. 10.4103/jpp.JPP_182_16 28405136 PMC5370329

[B15] Keating G. M. (2015). Netupitant/palonosetron: a review in the prevention of chemotherapy-induced nausea and vomiting. Drugs 75 (18), 2131–2141. 10.1007/s40265-015-0512-9 26613606

[B16] Ko K. R. Lee H. Han S.-H. Ahn W. Kim D. K. Kim I.-S. (2022). Substance P, a promising therapeutic target in musculoskeletal disorders. Int. J. Mol. Sci. 23 (5), 2583. 10.3390/ijms23052583 35269726 PMC8910130

[B17] Larusso J. Waldman S. A. Kraft W. K. (2008). Aprepitant for the prevention of nausea and vomiting associated with chemotherapy and postoperative recovery. Expert Rev. Clin. Pharmacol. 1 (1), 27–37. 10.1586/17512433.1.1.27 24410507

[B18] Ma P. Pan X. Liu R. Qu Y. Xie L. Xie J. (2022). Ocular adverse events associated with anti-VEGF therapy: a pharmacovigilance study of the FDA Adverse Event Reporting System (FAERS). Front. Pharmacol. 13, 1017889. 10.3389/fphar.2022.1017889 36467087 PMC9716077

[B19] Maru A. Gangadharan V. P. Desai C. J. Mohapatra R. K. Carides A. D. (2013). A phase 3, randomized, double-blind study of single-dose fosaprepitant for prevention of cisplatin-induced nausea and vomiting: results of an Indian population subanalysis. Indian J. Cancer 50 (4), 285–291. 10.4103/0019-509X.123580 24369195

[B20] Modi J. N. Cimino S. K. (2021). Incidence of ifosfamide induced encephalopathy in patients receiving concomitant fosaprepitant. J. Oncol. Pharm. Pract. 27 (8), 1891–1895. 10.1177/1078155220971794 33166244

[B21] Pojawa-Gołąb M. Jaworecka K. Reich A. (2019). NK-1 receptor antagonists and pruritus: review of current literature. Dermatology Ther. 9 (3), 391–405. 10.1007/s13555-019-0305-2 PMC6704190 31190215

[B22] Poli-Bigelli S. Rodrigues-Pereira J. Carides A. D. Julie Ma G. Eldridge K. Hipple A. (2003). Addition of the neurokinin 1 receptor antagonist aprepitant to standard antiemetic therapy improves control of chemotherapy-induced nausea and vomiting. Results from a randomized, double-blind, placebo-controlled trial in Latin America. Cancer 97 (12), 3090–3098. 10.1002/cncr.11433 12784346

[B23] Rojas C. Slusher B. S. (2015). Mechanisms and latest clinical studies of new NK1 receptor antagonists for chemotherapy-induced nausea and vomiting: rolapitant and NEPA (Netupitant/Palonosetron). Cancer Treat. Rev. 41 (10), 904–913. 10.1016/j.ctrv.2015.09.005 26442475

[B24] Schwartzberg L. S. Navari R. M. (2018). Safety of polysorbate 80 in the oncology setting. Adv. Ther. 35 (6), 754–767. 10.1007/s12325-018-0707-z 29796927 PMC6015121

[B25] Séjourné A. Noal S. Boone M. Bihan C. Sassier M. Andrejak M. (2014). Two cases of fatal encephalopathy related to ifosfamide: an adverse role of aprepitant? Case Rep. Oncol. 7 (3), 669–672. 10.1159/000368184 25408661 PMC4224236

[B26] Shirley M. (2021). Netupitant/palonosetron: a review in chemotherapy-induced nausea and vomiting. Drugs 81 (11), 1331–1342. 10.1007/s40265-021-01558-2 34292534 PMC8463343

[B27] Szabatura A. H. Cirrone F. Harris C. McDonnell A. M. Feng Y. Voit D. (2015). An assessment of risk factors associated with ifosfamide-induced encephalopathy in a large academic cancer center. J. Oncol. Pharm. Pract. 21 (3), 188–193. 10.1177/1078155214527143 24664476

[B28] Tsuda T. Kyomori C. Mizukami T. Taniyama T. Izawa N. Horie Y. (2016). Infusion site adverse events in breast cancer patients receiving highly emetic chemotherapy with prophylactic anti-emetic treatment with aprepitant and fosaprepitant: a retrospective comparison. Mol. Clin. Oncol. 4 (4), 603–606. 10.3892/mco.2016.769 27073673 PMC4812534

[B29] Vazirian F. Samadi S. Rahimi H. Sadeghi M. Mohammadpour A. H. (2022). Aprepitant, fosaprepitant and risk of ifosfamide-induced neurotoxicity: a systematic review. Cancer Chemother. Pharmacol. 90 (1), 1–6. 10.1007/s00280-022-04439-x 35635561

[B30] Xiong J. Zhao G. Yang S. Chen J. (2019). Efficacy, tolerability and pharmacokinetic impact of aprepitant in sarcoma patients receiving ifosfamide and doxorubicin chemotherapy: a randomized controlled trial. Adv. Ther. 36 (2), 355–364. 10.1007/s12325-018-0862-2 30607545

[B31] Xue F. Liu X. Qi X. Zhou J. Liu Y. (2023). The clinical research study for fosaprepitant to prevent chemotherapy-induced nausea and vomiting: a review. Adv. Clin. Exp. Med. 32 (6), 701–706. 10.17219/acem/157061 37026971

