# Supplementary material for: Safety assessment of neurokinin-1 receptor antagonist: real-world adverse event analysis from the FAERS database
Source: Front Pharmacol. 2024 Jul 31;15:1413709. doi: 10.3389/fphar.2024.1413709 (PMC11321956; doi:10.3389/fphar.2024.1413709)
Supplement: Supplementary file 3 [file Table1.DOCX]

2×2 contingency table

|  | Drug-related AEs | Non-drug-related AEs | Total |
| --- | --- | --- | --- |
| Drug | a | b | a + b |
| Non-drug | c | d | c + d |
| Total | a + c | b + d | N = a + b + c + d |

| Method | Formula | ﻿Threshold |
| --- | --- | --- |
| ROR | $ROR=\frac{a / c}{b / d}$ | a ≥ 3  ROR ≥ 3  ﻿95%CI (lower limit) > 1 |
|  | $SE(lnROR)=\sqrt{\frac{1}{a}+\frac{1}{b}+\frac{1}{c}+\frac{1}{d}}$ |  |
|  | $95\%CI= e^{\ln\left( ROR \right)\pm1.96se}$ |  |
| PRR | $PRR=\frac{a / (a+b)}{c / (c+d)}$ | a ≥ 3  PRR ≥ 2  ﻿95%CI (lower limit) > 1 |
|  | $SE(lnPRR)=\sqrt{\frac{1}{a}-\frac{1}{a+b}+\frac{1}{c}-\frac{1}{c+d}}$ |  |
|  | $95\%CI= e^{\ln\left( PRR \right)\pm1.96se}$ |  |
| BCPNN | $IC=\log_{2}\frac{p(x, y)}{p\left( x \right)p(y)}= {log}_{2}\frac{a(a+b+c+d)}{(a+b)(a+c)}$ | IC025>0 |
|  | $E(IC)=\log_{2}\frac{(a+\gamma11)(a+b+c+d+\alpha)(a+b+c+d+\beta)}{\left( a+b+c+d+\gamma\right)(a+b+\alpha1)(a+c+\beta1)}$ |  |
|  | $V\left( \mathrm{IC} \right)=\frac{1}{{(ln2)}^{2}}[\frac{\left( a+b+c+d \right)-a+\gamma-\gamma11}{\left( a+\gamma11 \right)\left( 1+a+b+c+d+\gamma\right)}+\frac{\left( a+b+c+d \right)-\left( a+b \right)+a-\alpha1}{\left( a+b+\alpha1 \right)\left( 1+a+b+c+d+\alpha\right)}+\frac{\left( a+b+c+d+\alpha\right)-\left( a+c \right)+\beta-\beta1}{\left( a+b+\beta1 \right)\left( 1+a+b+c+d+\beta\right)}]$ |  |
|  | $\gamma=\gamma11\frac{(a+b+c+d+\alpha)(a+b+c+d+\beta)}{\left( a+b+\alpha1 \right)(a+c+\beta1)}$ |  |
|  | $IC-2SD=E\left( \mathrm{IC} \right)-2 \sqrt{V(IC)}$ |  |
| EBGM | $EBGM=\frac{a(a+b+c+d)}{\left( a+c \right)(a+b)}$ | EBGM05>2 |
|  | $SE(lnEBGM)=\sqrt{\frac{1}{a}+\frac{1}{b}+\frac{1}{c}+\frac{1}{d}}$ |  |
|  | $95\%CI= e^{\ln\left( EBGM \right)\pm1.96se}$ |  |
